# Structural Proteins at Neuromuscular Junction Are Downgraded While NRG1 and Agrin Gene Expression Increases After Muscle Injury

**DOI:** 10.3390/biomedicines13092277

**Published:** 2025-09-16

**Authors:** Jurandyr Pimentel Neto, Lara Caetano Rocha-Braga, Matheus Bertanha Fior, Paula Oliveira Camargo, Adriano Polican Ciena

**Affiliations:** Laboratory of Morphology and Physical Activity (LAMAF), Institute of Biosciences (IB), São Paulo State University, Rio Claro 13506-900, São Paulo, Brazil; lara.rocha@unesp.br (L.C.R.-B.); matheus.b.fior@unesp.br (M.B.F.); paula.camargo@unesp.br (P.O.C.); adriano.ciena@unesp.br (A.P.C.)

**Keywords:** neuromuscular junction, muscle injury, bassoon, α-bungarotoxin, P/Q calcium channel

## Abstract

**Background/Objectives:** The neuromuscular junction (NMJ) is the area where peripheral nerves communicate with muscle tissue. Muscle injury can occur as part of an acute degenerative process at the NMJ. This study aims to investigate the remodeling of the NMJ after a muscle injury in an experimental model. **Methods:** We used sixty male Wistar rats divided into five groups: a control group (C) and four muscle injury groups (MI) at different time points: 0 h, 24 h, 48 h, and 7 d after injury. We subjected the right hind limb to muscle injury and dissected the gastrocnemius muscles for analysis. We employed light microscopy to examine cell nuclei and the connective tissue, immunostaining to identify and measure the pre- and postsynaptic regions as well as calcium channels (P/Q), and real-time PCR to assess the gene expression of NRG1 and Agrin. **Results:** Our findings revealed an accumulation of nuclei and connective tissue in the acute injury groups (0 to 48 h). The morpho-quantitative analyses showed that the presynaptic structures and calcium channels underwent similar remodeling due to their morpho-functional relationship. Meanwhile, the postsynaptic receptors were significantly affected by the degenerative and inflammatory processes. These results can be linked to increased expression of NRG1 and Agrin in the acute injury groups. **Conclusions:** In conclusion, the synaptic regions displayed substantial adaptations within the first 48 h, with the presynaptic region recovering rapidly and the postsynaptic region recovering slowly. This relationship suggests that significant increases in Agrin and NRG1 play a crucial role in maintaining the integrity of these structures.

## 1. Introduction

The neuromuscular junction (NMJ) is the microanatomical region of communication between the peripheral nervous system and muscle tissue. It mediates the signaling of the central nervous system and the somatic actions of the locomotor system resulting from muscle contraction, formed by three main components (a triad), namely the axon terminal, the sarcolemma of the muscle fibers, and the perisynaptic Schwann cells, which allow the transmission of neural impulses for muscle contraction, maintenance, and molecular support in the region [1,2,3].

The NMJ is addressed by various research groups, with advances in this area constantly disseminated and updated. Its aspects are analyzed regarding its plasticity and functionality, in response to aging, sarcopenia, degenerative diseases such as amyotrophic lateral sclerosis, spinal muscular atrophy, dystrophy, and physical activity or exercise, to evaluate its degeneration, maintenance, and development, as well as possible treatments for this region [4,5,6].

Given this context, NMJ adaptations directly and indirectly affect the muscle fibers and their pre- and post-synaptic structures, which can influence the pre- and post-receptors of these regions [7,8].

Regarding NMJ receptors, these are activated according to specific neurotransmitters (acetylcholine), which are essential for synapses at the NMJ, are released into the NMJ synaptic cleft, and interact with specific receptors (AChR), generating an action potential through the surface of the muscle fiber, which diffuses through the muscle tissue via the T tubules (sarcoplasmic reticulum). In this way, the action potential activates the opening and influx of calcium (Ca^2+^) into the cytoplasm through its specific channels. Ca^2+^ triggers the action of contractile proteins and, consequently, muscle contraction [9,10,11].

The functionality of the NMJ depends on metabolic signaling pathways. Agrin-LRp4-MuSK is one of the main pathways associated with the formation and maintenance of the NMJ, which participates in the functioning of synapses and the reinnervation process. Agrin is a protein expressed at the NMJ by nerve terminals, muscle fibers, and the CPS, and it is essential for the increase in AChR in muscle fibers [12]. The Agrin isoform produced by terminal axons is a potent factor in the promotion of AChR clusters, and muscle fibers also express Agrin at the NMJ. In addition, neuregulin 1 (NRG1) plays a fundamental role in development and maintenance after nerve injury, is essential for the process of repair and regeneration of the NMJ, and promotes the growth of nerve terminals and the formation of new junctions [13].

Skeletal striated muscle injuries are common in sports practices, but they have their own particularities regarding their level of severity. Thus, they can be classified as follows: tissue impairment worsening toward strain (muscle tissue involvement with an exacerbated extension of the region, without the extravasation of interstitial fluid), laceration (muscle tissue involvement with an exacerbated extension and a partial rupture of the fibers, with the extravasation of interstitial fluid), and contusion (muscle tissue involvement in the exacerbated extension of the region and complete rupture of the fibers of one or more muscle compartments, with the extravasation of interstitial fluid) [14,15].

The processes of muscle damage and repair are similar in most cases, as is the time of onset of the regenerative process; thus, they can directly or indirectly affect other structures and the functionality of the locomotor system [16,17]. The complications generated by muscle injury can result in muscle contracture, muscle atrophy, and fibrosis, which directly affect the biomechanics and functionality of the affected limb or region, in addition to compromising tissue volume and sensory and motor responses [15,18].

The process of muscle recovery following an acute injury begins with the formation of a fibrotic scar at the injury site. This scar formation is essential for the muscles to heal properly and regain their functionality. In many cases, specific treatments are necessary to ensure effective recovery [19,20].

Athletes, who rely on optimal muscle performance, may experience minor fibrosis that can still significantly impact their performance, even if the fibrosis is minimal. It is also important to recognize that recurring injuries often happen during physical activities [21,22], which can extend the recovery period [20].

Given the effects caused by muscle injury throughout the locomotor system, morphological and molecular analyses of the neuromuscular junction become viable, as well as the scope of its acute pre- and post-synaptic structural adaptation, thus establishing future perspectives regarding treatments for this essential junction.

## 2. Materials and Methods

### 2.1. Animals

We selected sixty male Wistar rats and divided them equally into five experimental groups. During the experimental period, the animals were allocated to cages (33 × 40 × 16 cm, *n* = 5) under the conditions of temperature monitoring (23 ± 2 °C) and a 12 h light/dark period, with food and water ad libitum. The procedures applied in this study were approved by the Committee on Ethics in Animal Use of the Institute of Biosciences of the São Paulo State University (CEUA—n°07/2022—approved on 14 September 2022).

### 2.2. Muscle Injury (MI) Protocol

The animals from the MI groups were previously anesthetized with intraperitoneal injection with doses standardized according to their weight with ketamine (90 mg/kg) and xylazine (10 mg/kg), placed in the prone position exposing the posterior surface of the right pelvic limb; in addition, local trichotomy was performed in the region referring to the animal’s triceps surae, where the muscle injury protocol was carried out.

In the MI protocol, we used a sterile hypodermic needle (22G × 1” and 0.70 mm × 25 mm) for each animal. To initiate the procedure, we cleaned the hind limb area, measured, and marked 2 cm above the animal’s calcaneus using a caliper. This designated region corresponds to the middle third of the gastrocnemius muscle. After that, we simulated the injury to induce local muscle distension, during which we inserted a hypodermic needle transversely into the gastrocnemius muscle [23].

### 2.3. Light Microscopy

We organized five samples per group from the belly of the gastrocnemius muscle, which were frozen in liquid nitrogen (−196 °C), and we subsequently took transverse sections (12 µm) stained with hematoxylin and eosin (HE) to highlight nuclei adaptation and Picrosirius Red (PS) for connective tissue staining [24,25]. For each sample, three histological slides were prepared from each animal for each respective staining, and three sections from the regions of interest were acquired from each slide.

The images of the sections were obtained using the Olympus^®^ BX51 (Shinjuku, Japan) optical light microscope from the Animal Cytogenetics Laboratory at UNESP Campus Rio Claro, SP, Brazil, using a 20× objective (200× magnification), where it was possible to qualitatively and quantitatively evaluate the inflammatory and fibrotic process through differentiation and an increase in cell nuclei and connective tissue.

### 2.4. Immunostaining—First Step

We prepared five samples per group in longitudinal sections (20 µm) (Cryostat HM 505E, MICROM^®^, Walldorf, Germany) for the immunostaining protocol. We collected the sections on histological slides, washed twice for 5 min with PBS, permeabilized with 0.1% Triton diluted in PBS for 10 min, and blocked with solution of 1% bovine serum albumin (BSA) also diluted in PBS for 20 min, then subsequently blocked with 5% of normal goat serum (NGS) diluted in PBS for 20 min.

### 2.5. Presynaptic Receptors (Bassoon) and Calcium Channels (P/Q)

After the immunostaining first step, we incubated the samples with the primary anti-Bassoon (1:500) to identify the presynaptic region and calcium channels (P/Q) overnight (16 h) at 4 °C [11]. Subsequently, the slides were washed for 2 × 5 min with PBS and incubated for 1 h with the secondary antibodies Alexa Fluor 488 (anti-rabbit) and Alexa Fluor 647 (anti-mouse) (1:1000) diluted in PBS containing 1% BSA, respectively. Immediately afterward, they were mounted with Prolong^®^ (Molecular Probes, Eugene, OR, USA) and stored in a freezer at –20 °C. We obtained the images using the Olympus^®^ BX61 (Shinjuku, Japan) Fluorescence Microscope of the Bacteria Genetics Laboratory (LGB), UNESP, Rio Claro, SP, Brazil.

### 2.6. Postsynaptic Receptors (AChR)

After the first step, we incubated the samples with anti-bungarotoxin, conjugated (Molecular Probes, Eugene, OR, USA), and diluted in PBS (phosphate-buffered saline) (1:600) to mark the postsynaptic region. Subsequently, we washed the slides (2 × 5 min) with PBS containing 1% BSA, mounted with Prolong^®^ (Molecular Probes, Eugene, OR, USA), and stored at –20 °C. After that, we obtained the images using the Olympus BX61^®^ (Shinjuku, Japan) Fluorescence Microscope of the Bacteria Genetics Laboratory (LGB), UNESP, Rio Claro, SP, Brazil [26].

### 2.7. Morphometry

We established the measurements of the total perimeter, stained perimeter, total area, stained area, and the dispersion of pre-synaptic receptors (Bassoon), calcium channels (P/Q), and postsynaptic receptors (α-bungarotoxin) [27]. We obtained a total of 10–12 images per animal for each region of interest/antibody, resulting in 50–60 images for each experimental group per region. We conducted the measurements using ImageJ^®^ (Version 1.54p, National Institutes of Health (NIH), Bethesda, MD, USA) software, and the statistical analysis using GraphPad Prism^®^ 8.4.3 software [28,29].

The measurement process began with the following steps for each selected image:

(A) Before starting the measurements, we selected the “Straight” option, and we drew a line on the scale bar (5 μm), standardized on a single microscope with images magnified to 1000×. In the Analyze/Set Scale tab, we set the Known Distance value to 5 and adjusted the measurement unit to μm.

(B) To measure the stained area and stained perimeter, we accessed the Analyze/Set Measurements tab, selecting the “Area” and “Perimeter” options. The next step involved navigating to the Process/Binary/Make Binary tab to process the area displayed by the software. By clicking on the Edit/Create Selection tab and pressing the M key, the area and perimeter values were generated by the software in a new window according to the pre-calculated data. We recorded these values in a spreadsheet for subsequent statistical analysis.

(C) The procedure for calculating the total area and total perimeter was the same as that for the stained area and perimeter. Before beginning the binarization process, we accessed the Image/Type/RGB Stack tab. Then, the Adjustment/Threshold option under the Image tab standardizes the area according to the image, highlighting the entire region from the junction. The following steps were like those in section B. 

(D) To measure the maximum diameter, we made prior adjustments in the Analyze/Set Measurements tab. We first selected the line option and crossed diagonally the extremes of the highlighted region using the Straight tool. We drew a line from one end to the other, and by pressing the M key, a window displayed the diameter length, which we recorded in the spreadsheet for analysis [29].

Additionally, we calculated the dispersion value using the pre-calculated values of the stained area and the total area. The formula used was the stained area divided by the total area, multiplied by one hundred [7]. This formula was pre-set in the data-recording spreadsheet to automatically calculate the result as soon as we entered the values, and we recorded the final result as a percentage.

### 2.8. Real-Time PCR

We used the gastrocnemius muscle of five animals from each experimental group in this study. We macerated and homogenized the samples using a manual macerator (mortar and pestle) to collect the tissue while preventing errors and potential contamination. We duplicated all samples with the homogenized material divided into two Eppendorf tubes corresponding to each sample from the five animals.

Next, we diluted the samples in 500 µL of Trizol reagent (Thermo Fisher Scientific, Invitrogen^®^, Waltham, MA, USA) until complete solubilization occurred. After that, we incubated the mixture at room temperature for 5 min with the addition of 150 µL of chloroform (LABSYNTH, São Paulo, Brazil) for deproteinization. Following this, we used a centrifuge to separate the supernatant at 12,000× *g* for 15 min at 4 °C, and we precipitated the RNA in the aqueous phase using isopropanol (LABSYNTH, São Paulo, Brazil). We centrifugated the samples again at 12,000× *g* for 10 min at 4 °C, washed with 75% ethanol (LABSYNTH, São Paulo, Brazil), and finally, dissolved in 60 µL of deionized H_2_O (Life Technologies, Carlsbad, CA, USA). After the extraction process, we stored the RNA samples in a freezer at –80 °C.

We measured the RNA concentration at a wavelength of 260 nm, and the purity was determined using the 260/280 ratio (~1.9) with a Agilent BioTek^®^ Synergy™ H1 multi-Mode Reader spectrophotometer (Agilent Technologies, Inc., Santa Clara, CA, USA) and the Gen5™ Data Analysis Software, using 0.2 mL per sample. We conducted the extraction process in the Department of Biodiversity at UNESP, Campus Rio Claro, SP, and the sample concentration at the Laboratory of Bacterial Genetics (LGB), UNESP, Rio Claro, SP, Brazil.

In addition, we conducted the quantitative analysis of gene expression using a thermocycler (QuantStudio 3 Real-Time PCR, Thermo Scientific, Waltham, MA, USA). We selected and designed the oligonucleotides using data and references from the National Center for Biotechnology Information (NCBI—https://www.ncbi.nlm.nih.gov/—accessed on 21 November 2024), and we used the following oligonucleotides (forward [F], reverse [R], and NCBI code [NM]) for the gene expression analysis:

GAPDH: F: CATCACTGCCACCCAGAAGACTG, R:ATGCCAGTGAGCTTCCCGTTCAG (NM_017008.4)—normalizer.

β-Actin: F: CCCAGGCATTGCTGACAGG, R:ATAGAGCCACCAATCCACACAG (NM_031144.3)—normalizer.

Agrin: F: GAGATCCTCAACGTGGACCC, R:TTGCCTTTCAAGTACCGCCA (NM_001429555.1).

NRG1: F: AGTCAGGAACTCAGCCACAA, R:CCAGTCGTGGATGTCGATGT (NM_001271118.3).

The assays were performed with the solutions contained in the GoTaq^®^ 1-Step RT-qPCR System kit (A6020—Promega Corporation, Fitchburg, WI, USA), organized in duplicates in the plates (96 wells), where 4 µL of synthesized RNA was pipetted per well, added to the reaction mixture containing 10 µL of GoTaq^®^ qPCR Master Mix 2x (Promega Corporation, WI, USA), 2 µL of ultra-pure water (nuclease-free water), and 2 µL (100 NM) of each primer (forward and reverse). The amplification parameters were set according to the One-Step kit manual [30] following the parameters below to obtain the curves:

(1) Reverse Transcriptase (RT)—1 cycle 37 °C for 60 min.

(2) RT Inactivation and Heating Activation—1 cycle at 95 °C for 15 min.

(3) 40 cycles.

(3.1) Denaturation—95 °C for 30 s.

(3.2) Annealing—58 °C for 45 s.

(3.3) Extension—72 °C for 1 min.

(4) Dissociation—1 cycle at 60–95 °C.

At the conclusion of the steps, we compared the quantification threshold (Ct) for each sample between duplicates and automatically determined by the software. We normalized the expression levels of the target genes to the levels of GAPDH and β-actin, which served as endogenous controls within each group. We calculated the fold-change values and organized them in a spreadsheet to determine the means and standard deviations for each primer by group.

We conducted the quantitative analysis at the Industrial Microbiology Laboratory of the Institute of Biosciences, UNESP, in Rio Claro, SP, under the supervision of Prof. Dr. Jonas Contiero.

For the statistical analysis, we performed the calculation of means and standard deviations, conducted a one-way ANOVA test, accompanied by Tukey’s post hoc test for comparing groups for each primer of interest, and established a significance level of *p* < 0.05 using GraphPad Prism^®^ version 8.4.3.

## 3. Results

### 3.1. Light Microscopy

**Figure 1:**
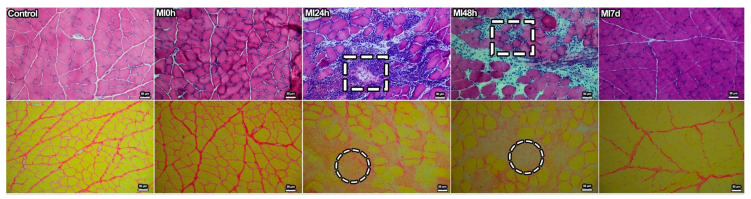
(HE) Light microscopy with hematoxylin and eosin staining to demonstrate the change in nuclear arrangement in all muscle groups that presented high regionalized nuclear concentration, in the 24 h and 48 h periods (dashed square) given the regenerative process of muscle fibers, already renewed in the 7d period. Bars: 50 µm. (PS) Light microscopy with Picrosirius staining, where it was possible to demonstrate the aspects of the connective tissue in all experimental groups by periods, which presented regions of accumulation of associated connective tissue between the fibers and regions of fibrosis as an inflammatory effect in the face of the muscle injury protocol (dashed circle). Bars: 50 µm.

We stained the cross-sections of muscle fibers with HE and analyzed those using a light microscope to qualitatively assess tissue involvement and reveal adaptations in different periods (Figure 1-HE). In Group C, it was possible to observe the nuclei peripheral to the muscle fibers in a standard aspect, corresponding to the tissue morphology of the skeletal striated muscle of the animals. In Group MI0h, we observed an increase in peripheral nuclei and rearrangement of muscle fibers with a reduction in the interstitial limits between them. In Group MI24h, it was possible to identify the injured region and a massive accumulation of cell nuclei and fibers with centralized nuclei. In Group MI48h, the muscle fibers already show signs of regeneration with circular aspects, and the nuclei in the region show a lower density compared to Group MI24h. Finally, Group MI7d presents the standardized aspect of the circular fibers, still with additional nuclei in the interstitial tissue.

We used the cross-sections of the region for qualitative evaluation of the morphology of the connective tissue between the fibers and to identify the tissue fibrosis. For this purpose, we stained them with PS (Figure 1-PS). In Group C, it was possible to observe connective tissue associated with the muscle fibers and the arrangement and congruence of these fibers. In Group MI0h, it was possible to identify the increase in interstitial collagen between the muscle fibers without changes in the muscle tissue architecture. The MI24h Group showed the first signs of connective tissue accumulation and a fibrotic aspect, with the muscle fibers mischaracterized in the affected region. In comparison, in Group MI48h, it was possible to observe fibrosis and the rearrangement of muscle fibers in a state of muscle regeneration as a post-inflammatory effect. Finally, in Group MI7d, the reconstruction of the muscle fibers was observed in a standard arrangement but with a reduction in the connective tissue between them compared to Group C.

### 3.2. Presynaptic Region (Bassoon)

**Figure 2:**
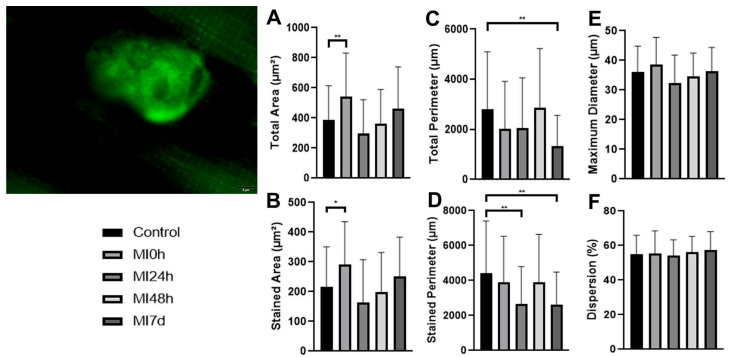
Qualitative image of the presynaptic active zone (Basson). Bar: 5 µm. Means and standard deviations of the measurements of (**A**) total area—C≠MI0h (*p* < 0.01) **; (**B**) stained area—C≠MI0h (*p* < 0.05) *; (**C**) total perimeter—C≠MI7d (*p* < 0.01) **; (**D**) stained perimeter—C≠MI24h and C≠MI7d (*p* < 0.01) **; (**E**) maximum diameter; and (**F**) dispersion of the presynaptic receptors of all experimental groups.

In the presynaptic identification, we used longitudinal sections of the gastrocnemius muscle, and we realized the incubations (anti-Bassoon), and subsequently, the measurements of the images (Figure 2).

The total area of the MI0h Group compared to the C Group presented average values 40.3% larger (*p* < 0.01); in turn, the MI24h Group was 23.9% smaller, the MI48h Group 6.7% smaller, and the MI7d Group 29.7% larger. Between the post-injury periods, compared to the MI0h Group, MI24h presented a value 45.7% lower, MI48h 33.5% lower, and MI7d 14.6% lower compared to the MI24h Group, the MI48h Group obtained an average 22.4% higher and MI7d 57.3% higher, and finally, between MI48h and MI7d, there was an increase of 28.4% (Figure 2A).

In the stained area values, the MI0h Group presented a mean 34.8% higher (*p* < 0.05), MI24h 2.4% lower, MI48h 7.3% lower, and MI7d 16.6% higher than the C Group. In comparison with MI0h, MI24h presented a mean 43.6% lower and MI48h 31.3% lower; the MI7d Group presented a mean value 13.4% lower. About MI24h, the MI48h group presented a 21.9% higher value, and MI7d 53.6% higher. In the last comparison, MI48h presented a value 25.9% higher than MI7d (Figure 2B)

About the total perimeter, the MI0h Group presented an average 27.7% lower (*p* < 0.05), MI24h 26.9% lower, MI48h 2.3% higher, and MI7d 52.8% lower than the C Group; between the periods, MI24h presented an average 1.1% higher, MI48h 41.7% higher, and MI7d 34.6% lower compared to the MI0h Group; compared to the MI24h Group, MI48h presented a value 40.1% higher and MI7d 64.4% higher; and between MI48h and MI7d, the average was 116.9% lower for the respective variable (Figure 2C).

In the stained perimeter, the MI0h Group showed mean values that were 11.5% lower than the C Group. The MI24h Group had mean values that were 39.4% lower (*p* < 0.01), while the MI48h Group was 11.3% lower and the MI7d Group was 40.2% lower (*p* < 0.01). Between the post-injury periods, compared to the MI0h Group, MI24h presented a value of 31.5% lower, MI48h 0.4% higher, and MI7d 32.4% lower. Compared to the MI24h Group, the MI48h Group obtained a mean 46.7% higher, and the MI7d Group 1.3% lower. Finally, between MI48h and MI7d, there was a reduction in the mean value of 32.7% (Figure 2D).

Regarding the maximum diameter, the MI0h Group presented an average 6.9% larger, MI24h 9.8% smaller, MI48h 3.6% smaller, and MI7d 1.2% larger when compared to the C Group. MI24h presented an average 15.6% smaller, MI48h 9.9% smaller, and MI7d 5.3% smaller compared to the MI0h Group; compared to the MI24h Group, MI48h presented a value 6.8% larger and MI7d 12.2% larger; and between MI48h and MI7d, the average was 5% higher for the variable (Figure 2E).

In the dispersion variable, the MI0h Group presented an average value 1.07% higher, MI24h 1.5% smaller, MI48h 2% larger, and MI7d 4.6% higher than the C Group. In comparison with MI0h, MI24h presented a mean of 2.6% lower, and MI48h 0.9% higher; meanwhile, the MI7d Group presented a mean value of 3.4% higher. About MI24h, the MI48h Group presented a value of 3.6% and MI7d 6.2% higher. Finally, in the last comparison between MI48h and MI7d, the MI7d Group presented a mean value of 2.5% higher (Figure 2F).

### 3.3. Calcium Channel (P/Q)

**Figure 3:**
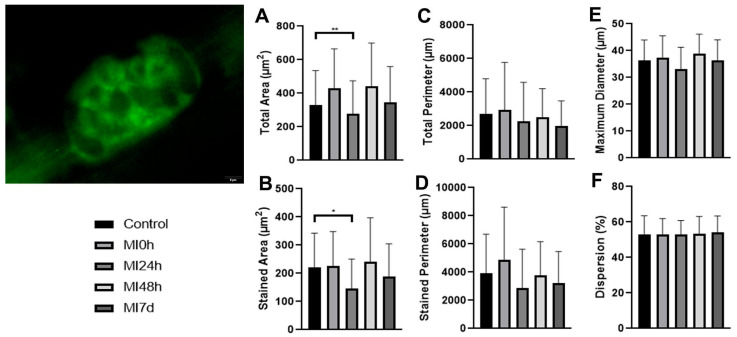
Immunostaining image of the P/Q calcium channels in the neuromuscular junction. Bar: 5 µm. Means and standard deviations of the measurements of (**A**) total area—C≠MI24h (*p* < 0.01) **; (**B**) stained area—C≠MI24h (*p* < 0.05) *; (**C**) total perimeter; (**D**) stained perimeter; (**E**) maximum diameter; (**F**) dispersion of calcium channels (P/Q) in the experimental groups.

Longitudinal sections of the gastrocnemius muscle were prepared and incubated with the P/Q calcium channel antibody. Afterward, we captured the images and measured them to obtain the means and standard deviations of the variables (Figure 3).

About the total area, in comparison with the C5 Group, the MI0h Group presented average values 2.1% higher, the MI24h Group 33.7% lower (*p* < 0.01), the MI48h Group 5.2% higher, and the MI7d Group 17.3% lower. Among the post-injury periods, compared to the MI0h Group, MI24h presented a 35% lower value, MI48h 0.88% higher, and MI7d 19% lower; compared to the MI24h Group, the MI48h Group obtained an average 58.3% higher and MI7d 24.6% higher; finally, between MI48h and MI7d, there was a reduction of 19.7% (Figure 3A).

The MI0h Group stained area presented a mean 1.9% higher, MI24h 33.8% lower (*p* < 0.05), MI48h 8.6% higher, and MI7d 14.6% lower than the C Group. In comparison with MI0h, MI24h presented a mean of 35.1% lower, and MI48h 6.5% higher, and the MI7d Group presented a mean value of 16.3% lower. About MI24h, the MI48h group presented a value 64.3% higher, and MI7d 29% higher. In the last comparison, MI48h presented a value 14.6% lower than MI7d (Figure 3B)

About the total perimeter, the MI0h Group presented an average 9.2% higher, MI24h 16% lower, MI48h 7.5% lower, and MI7d 26.7% lower than the C Group; between the periods, MI24h presented an average 23.1% lower, MI48h 15.3% lower, and MI7d 32.9% lower compared to the MI0h Group; compared to the MI24h Group, MI48h presented a value 10.1% higher and MI7d 12.7% lower; and between MI48h and MI7d, the average was 20.8% lower for the respective variable (Figure 3C).

The C Group stained perimeter was compared to the MI0h Group, which presented mean values 24.7% higher, the MI24h Group 26.5% lower, the MI48h Group 3.5% lower, and the MI7d Group 17.3% lower. Between the post-injury periods, compared to the MI0h Group, MI24h presented a value 41.4% lower, MI48h 22.6%, lower and MI7d 33.7% lower compared to the MI24h Group; the MI48h Group obtained a mean 31.4% higher and MI7d 12.5% higher; and finally, between MI48h and MI7d, there was a reduction in the mean value of 14.3% (Figure 3D).

Regarding the maximum diameter, the MI0h Group presented an average 2.8% larger, MI24h 9.3% smaller, MI48h 6.4% larger, and MI7d 0.05% smaller when compared to the C Group. MI24h presented an average 11.8% smaller, MI48h 3.4% larger, and MI7d 2.8% smaller compared to the MI0h Group; compared to the MI24h Group, MI48h presented a value 17.4% larger and MI7d 10.2% larger; and between MI48h and MI7d, the average was 6.1% smaller for the variable of interest (Figure 3E).

Lastly, the dispersion of the MI0h Group did not present a different mean value than the C Group, but about the other groups, MI24h was 0.3% lower, MI48h was 0.7% higher, and MI7d was 2% higher than the C Group. In comparison with MI0h, MI24h presented a mean 0.3% lower, and MI48h was 0.7% higher; meanwhile, the MI7d Group presented a mean value of 2% higher. About MI24h, the MI48h group presented a value of 1% and the MI7d 2.3% higher. In the last comparison, MI48h showed a mean value 1.3% higher than the MI7d Group (Figure 3F).

### 3.4. Postsynaptic Receptors (AChR)

**Figure 4:**
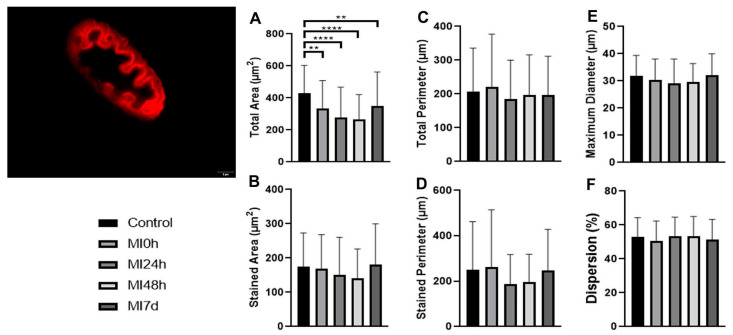
Immunostaining image of the postsynaptic receptors. Bar: 5 µm. Means and standard deviations of measurements of (**A**) total area—C≠MI0h, C≠MI7d (*p* < 0.01) ** and C≠MI24h, C≠MI8h (*p* < 0.0001) ****; (**B**) stained area; (**C**) total perimeter; (**D**) stained perimeter; (**E**) maximum diameter; (**F**) dispersion of postsynaptic receptors (AChR) in all experimental groups.

To identify the acetylcholine receptors in the postsynaptic region, we prepared longitudinal sections of the gastrocnemius muscle, followed by incubations and detailed photomicrographs, enabling comprehensive measurements of the proposed variables across all experimental groups (Figure 4).

Given the calculations of the total area variable in comparison with the C Group, the MI0h Group presented mean values 22.1% lower (*p* < 0.01), the MI24h Group 35.3% lower (*p* < 0.0001), the MI48h Group 37.7% lower (*p* < 0.0001), and the MI7d Group 18.7% lower (*p* < 0.01). Between the post-injury periods compared to the MI0h Group, MI24h presented a value 16.9% lower, MI48h 19.9% lower, and MI7d 2.8% higher compared to the MI24h Group, the MI48h Group obtained an average 3.5% lower and MI7d 25.7% higher, and finally, between MI48h and MI7d, there was an increase of 30.4% (Figure 4A).

Regarding the stained area, the MI0h Group presented an average 3.6% lower, MI24h 13.1% lower, MI48h 19.2% lower, and MI7d 3.2% higher than the C Group. Between the periods, MI24h presented an average 9.7% lower, MI48h 16.2% lower, and MI7d 7.1% higher compared to the MI0h Group. Compared to the MI24h Group, MI48h presented a value of 7.1% lower, and MI7d 18.7% higher. Between MI48h and MI7d, the average was 27.9% higher for the respective variable (Figure 4B).

The MI0h Group total perimeter presented an average 6.8% higher than the C Group, MI24h 11.2% lower, MI48h 5.3% lower, and MI7d 5.9% lower. In comparison with MI0h, MI24h presented an average 16.8% lower, MI48h 11.3% lower, and MI7d 11.5% lower. About MI24h, the MI48h group presented a value 6.6% higher and MI7d 6.4% higher. In the last comparison, MI48h presented a value 0.1% lower than MI7d (Figure 4C).

The stained perimeter of the MI0h Group presented mean values 4.9% higher than the C group, the MI24h Group 25.5% lower, the MI48h Group 21.1% lower, and the MI7d Group 1.1% lower than the C group. Between the post-injury periods, compared to the MI0h Group, MI24h presented a value 29.1% lower, MI48h 24.7% lower, and MI7d 5.7% lower compared to the MI24h Group, the MI48h Group obtained a mean 6.1% higher and MI7d 32.7% higher, and finally, between MI48h and MI7d, there was an increase in the mean value of 25.1% (Figure 4D).

Regarding the maximum diameter, the MI0h Group presented an average 4.9%, MI24h 8.4%, and MI48h 6.9% smaller than the C Group, while MI7d was 0.3% larger. Between the periods, MI24h presented an average 3.7% smaller, MI48h 2.1% smaller, and MI7d 5.5% smaller compared to the MI0h Group. Compared to the MI24h Group, MI48h presented a value 1.7% larger and MI7d 9.7% larger. Between MI48h and MI7d, the average was 7.8% larger than the respective variable (Figure 4E).

Lastly, for the dispersion variable, the MI0h Group presented an average 5.4% smaller, MI24h 0.7% smaller, MI48h 0.6% larger, and MI7d 3.2% smaller than the C Group. In comparison with MI0h, MI24h presented a mean 5.9% higher, and MI48h 5.7% lower; the MI7d Group presented a mean value 1.6% higher. About MI24h, the MI48h group presented a value of 0.1% lower and MI7d 4.2% lower. In the last comparison, MI48h presented a mean value 3.8% lower than the MI7d Group (Figure 4F).

### 3.5. PCR in Real Time

**Figure 5:**
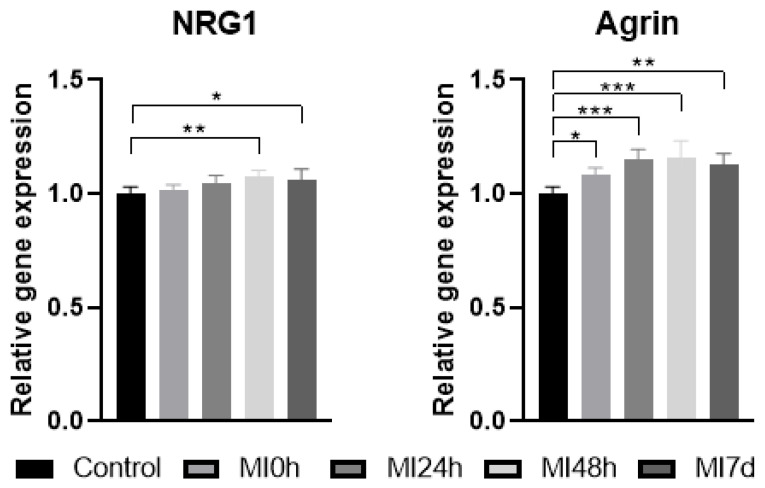
Graphs of the values obtained for relative gene expression of the oligos of interest (NRG1 and Agrin) related to the experimental groups. NRG1: C≠MI48h (*p* < 0.01) **, C≠MI7d (*p* < 0.05) *; Agrin: C≠MI0h (*p* < 0.05) *, C≠MI24h and C≠MI48h (*p* < 0.001) ***, C≠MI7d (*p* < 0.01) **.

Regarding the data obtained, we defined the GAPDH and β-Actin normalizers to standardize the analysis of the oligos of interest (Figure 5) using the ΔΔCT.

The expression values of the NRG1 gene showed a progressive increase in the post-injury groups MI0h, MI24h, and MI48h (*p* < 0.01) and a brief reduction in the MI7d Group (*p* < 0.05), but still higher than the C, MI0h, and MI24h groups.

The gene expression of Agrin showed a progressive increase in the periods of 0 h (*p* < 0.05), 24 h (*p* < 0.001), and 48 h (*p* < 0.001), and a reduction in Group MI7d (*p* < 0.01), with expression values closer to the Control, but still higher than in the MI0h group.

## 4. Discussion

The results revealed the acute effects of muscle injury in an experimental model, by presenting its qualitative repercussions about the arrangement of cell nuclei, formation of fibrotic regions, and consequently, regionalized nuclear concentration, which was proved quantitatively with analysis of the volume of these variables. In addition, it was possible to observe the maintenance of postsynaptic receptors and calcium channels with rapid structural recovery and the degenerative repercussion of postsynaptic receptors (AChR) that, even after 7 days, did not return to their normal appearance.

Deschenes et al. [31] demonstrated in the plantar, soleus, and long extensor muscles of the fingers in a C group the isolated measurements of presynaptic variables (Basson) and calcium channels (P/Q) and their relationship with the use of double labeling through the immunofluorescence technique, to present their relationship and possibility of individual and joint analysis and determine their relevance regarding presynaptic adaptation. Therefore, our study was adapted to individually demonstrate the repercussions of these structures in the process of muscle injury.

Regarding the morphological aspects and variables of the presynaptic receptors analyzed through measurements of the images obtained with immunostaining of the anti-Bassoon antibody, we were able to observe an increase in the receptor area in Group MI0h with a reduction in subsequent periods, until the return to a conformation close to C in Group MI7d. This may determine the rapid adaptation to the deleterious stimulus, as well as the rapid maintenance of this region. That contributes to the opening of the channels in this region, as described by Block-Gallego et al. [32] and associated with the calcium channels (P/Q) analyzed. which also presented rapid adaptation and similar changes. Such adaptations are reported by Deschenes et al. [31] as necessary due to the dependence on these considered active zones for the release of vesicles (Ach), and consequently, the return of the transmission of nerve impulses that, if they do not occur, can compromise tissue functionality.

Regarding the perimeter, we found a distinction between the structures that presented reductions at 0 h and 24 h, rearrangement at 48 h, and compaction at 7 d. that is, the area of these receptors was compromised in the acute period, but their structure was maintained in the face of their involvement. This is related to the data obtained on the diameter and dispersion of the analyzed region, which showed no significant differences regarding the variables analyzed statistically. That result is different from that found in an experimental model of motorized treadmill training, which considered endurance training (performed for 10 weeks) in old and young rats, which presented a considerable increase in this variable [33]. Meanwhile, it aligns with disuse in a hindlimb suspension model used to check this condition in the face of muscle atrophy, where this variable remained practically even in the face of the protocol conditions in the soleus, plantar, and long extensor muscles of the fingers [34].

The reduction in this kind of active zone, where the presynaptic receptors are located, occurs with aging. This change in the number of active zones is precipitated by the selective degeneration of the proteins that make up the active zones [35,36] because of degenerating axonal transport [37]. Thus, the age range of the experimental model may have contributed to rapid regeneration, as well as the fact that we directly applied the injury protocol to the muscle tissue.

The calcium channels showed a reduction in their area, perimeter, and diameter, albeit subtly, over the 24 h period. However, at 48 h after the injury, their structure and the area composed of receptors had practically returned to their standard conformation, demonstrating their high capacity for reorganization to maintain not only the structure but also the functionality of these channels, which are of utmost importance for responses to stimuli that constantly influence the neuromuscular system, as described by Day et al. [38] in a study about the influx and efflux of calcium for the transmission of nerve impulses from the axon terminal to the sarcolemma of the muscle fiber.

The calcium channels (P/Q), analyzed in this study, function as high-voltage-dependent calcium channels and contribute to the exocytosis of ACh vesicles in the synaptic terminals [39]. In our results, the structure of the channels maintained a structural dispersion about the analyzed variable similar to that resulting from the dispersion of presynaptic receptors (Bassoon), which once again demonstrates their structural, functional, and molecular relationship, as already described by Nishimune et al. [40], and consequently, their dependence on the region where they are found [41].

Postsynaptic receptors (AChR) were the most compromised structures in the proposed injury protocol due to direct injury in the muscle belly region, where they are located. That they are the most compromised was already evidenced by Yin et al. [42] and has been morphologically, quantitatively verified in our study.

The receptors showed a progressive reduction in their area up to MI48h, and even after MI7d, they did not return to the patterns found in the C Group, due to the extensive impairment established. This held true even in the face of the regenerative process in the muscle tissue, which directly affects the structure of the sarcolemma. This was already presented in the literature in healthy animals compared to a muscular dystrophy model, and in a muscular dystrophy model associated with a muscle injury that, in the face of progressive degeneration, presented a significant reduction in the structure of the NMJ about the involvement of the analyzed muscle [43,44].

The results obtained regarding the morphological variables of the AChR receptors revealed the impairment of this region in the most acute periods, a determining factor for the observation of the reduction in postsynaptic functionality in this recovery period, resulting mainly from the impairment already observed in the membranes present at the NMJ, which impact local signaling and the specific channels affecting the motor apparatus nerve impulses [45,46,47].

Postsynaptic receptors present high plasticity amid the repercussions facing the analyzed muscle tissue, which alters the reception of AChR vesicles that allow muscle contraction. This was already observed in endurance training protocols [27]; resistance training with a vertical jump [48]; resistance training with a load or without a load and associated [28]; and an experimental model of stretching and swimming [49], mainly with the organization and rearrangement of these structures. However, in the face of the degenerative process, this region tries to reduce or increase dispersion, as already observed in an immobilization model [50].

The functionality of the NMJ depends on metabolic signaling pathways like the Agrin-Lrp4-MuSK pathway, which is associated with the formation, functioning, and maintenance of synapses. Agrin is a protein expressed at the NMJ and is essential for the increase in AChR in muscle fibers [12]. The Agrin isoform is essential in promoting AChR clusters. In addition, NRG1 plays a fundamental role in the development and maintenance of the NMJ and is essential for the process of repair and regeneration of the region [13]. These facts corroborate the data presented on relative gene expression with a progressive increase in Agrin and NRG1 in the period from 0 h to 48 h, which may be related to the reduction in postsynaptic protein receptors in the same period, as presented by immunostaining, a technique which may determine the molecular response of these genes to the maintenance of receptors in the face of this type of muscle injury.

This study’s limitations include the analysis of specific genes associated with the morphological adaptations of the synaptic regions of the NMJ. Future studies should explore more genes, such as acetylcholine subunits (alpha1, alpha7, beta, gamma, epsilon), MUSK, LRP4, and others, associated with the NMJ pathways. These genes can be associated with different stimuli that promote plasticity and can reveal more about the NMJ recovery process following muscle injury. We chose the gastrocnemius muscle due to the standardization of the protocol, as it is the most affected and easily accessible for sample collection. Furthermore, future studies should explore different muscles, such as the plantaris, soleus, biceps, and triceps brachii, to evaluate and compare the repercussions of distinct types of muscle injuries in acute and chronic periods.

## 5. Conclusions

We conclude that the structure of the pre- and postsynaptic neuromuscular junction showed a reduction in the acute period, according to the measurements associated with the proteins of each region and the calcium channels responsible for the functioning of the synapse. In addition, the reduction in the most drastic period, from 0 h to 48 h, was inversely proportional to the NRG1 and Agrin genes that act in the maintenance of synaptic receptors, which showed a significant increase in this same period. This data can determine their molecular relationship in the face of muscle injury, and it points to the need for these genes for the structural and functional regenerative process that occurs after 7 days in the pre- and postsynaptic structures. Based on these innovative results about the acute injury protocol, we recommend that future research should explore more genes linked to morphological features, specifically their connection to diseases and the processes of degeneration and regeneration in the muscle injury process.

## Data Availability

All relevant data is contained within the article: The original contributions presented in this study are included in the article, and further inquiries can be directed to the corresponding author.

## Abbreviations

The following abbreviations are used in this manuscript:

AChR	Acetylcholine Receptors
BSA	Bovine Serum Albumin
NGS	Normal Goat Serum
NMJ	Neuromuscular Junction
PBS	Phosphate-Buffered Saline
